# Recent Progress on Integrated Energy Conversion and Storage Systems

**DOI:** 10.1002/advs.201700104

**Published:** 2017-05-17

**Authors:** Bin Luo, Delai Ye, Lianzhou Wang

**Affiliations:** ^1^ Nanomaterials Centre School of Chemical Engineering and Australian Institute for Bioengineering and Nanotechnology The University of Queensland St Lucia QLD 4072 Australia

**Keywords:** integrated energy conversion, integrated energy storage, mechanical energy, solar energy, thermal energy

## Abstract

Over the last few decades, there has been increasing interest in the design and construction of integrated energy conversion and storage systems (IECSSs) that can simultaneously capture and store various forms of energies from nature. A large number of IECSSs have been developed with different combination of energy conversion technologies such as solar cells, mechanical generators and thermoelectric generators and energy storage devices such as rechargeable batteries and supercapacitors. This review summarizes the recent advancements to date of IECSSs based on different energy sources including solar, mechanical, thermal as well as multiple types of energies, with a special focus on the system configuration and working mechanism. With the rapid development of new energy conversion and storage technologies, innovative high performance IECSSs are of high expectation to be realised for diverse practical applications in the near future.

## 1 Introduction

Energy shortage and environmental deterioration resulting from insufficient fossil fuel supplies and increasing consumption has becoming two major global problems for human beings.^1^ Developing new technology to make full use of the abundant “green” energies in the forms of solar, mechanical, and thermal energies have been recognised as a promising and effective way for our long‐term energy needs and environmental sustainable development.^2^ Over the past few decades, a large number of energy conversion technologies such as solar cells,^3^ mechanical generators,^4^ and thermoelectric generators,^5^ have been developed to convert the “green” energies into electrical energy, which is the most widely used energy type in our current society.[[qv: 2b,6]] However, the major drawback of these “green” energies is that electricity generation is highly dependent on the availability of the energy sources (e.g., sunlight, wind, heat), which is always not in good alignment with the actual demand.

One promising solution is to develop an integrated energy conversion and storage system (IECSS) that can simultaneously capture energy from the environment and store it with effective electrochemical energy storage devices for future energy demands.^7^ A variety of electrochemical energy storage devices including rechargeable batteries^8^ (e.g., lithium‐ion batteries (LIBs), lithium‐oxygen (Li‐O_2_) batteries, lithium‐sulfur batteries, redox flow batteries (RFBs)) and supercapacitors^9^ are the options for this purpose. For example, solar energy has been recognised as one of most abundant renewable energy sources.[[qv: 2c,3]] Many new conceptual solar energy based IECSSs like solar batteries or solar capacitors, have been proposed for solar energy storage.[[qv: 7b,d]] Under the sunlight illumination, a photo‐charging process will convert the solar energy into electrical energy and store it through an electrochemical way; the stored electrochemical energy can then be discharged as electric power output for electronics. Compared with other solar fuel generation approaches where new chemical bonds are formed, the solar energy based IECSSs may take benefits from the efficient and economic storage as well as minimized energy loss during discharging. Meanwhile, the development of IECSSs based on other types of green energies, i.e. mechanical and thermal energy, will also bring promising power choices for future electronic devices.

In the past few years, there have been many excellent reviews on the energy conversion (solar cells,^10^ photocatalysis,^11^ nanogenerators,^4^, ^12^ thermoelectric generators^13^) and electrochemical energy storage technologies (rechargeable batteries[[qv: 8a,14]] or supercapacitors[[qv: 9a,b,15]]), and also some articles that discussed some types of IECSSs, like photo‐rechargeable systems,[[qv: 7b,d]] self‐powered nanosystems,^16^ hybrid energy cells for harvesting multi‐mode energies.^17^ Encouragingly, some innvoative IECSSs that can photo‐ or electro‐ chemically convert renewable energies into other energy sources such as valuable chemical fuels.^18^ For example, new types of two‐dimensional materials and their heterostructures have recently been developed as efficient electrocatalysts or photoelectrocatalysts to effectively promote clean fuel hydrogen evolution.^19^ Nevertheless, with the rapid development of energy conversion and storage technologies,[[qv: 8b,10,20]] a comprehensive review covering on the design and assembly of IECSSs for different energy source is very important to provide researchers better understanding on the progress in this burgeoning field. This review summarizes the recent progress of IECSSs that could effectively capture the energy generated from solar, mechanical, thermal as well as multiple energy sources, with emphasis on their component and function integration and related working mechanism. A brief summary of current status and perspectives of IECSSs will also be provided for future research directions.

## 2 Solar Energy Based IECSSs

**Figure 1:**
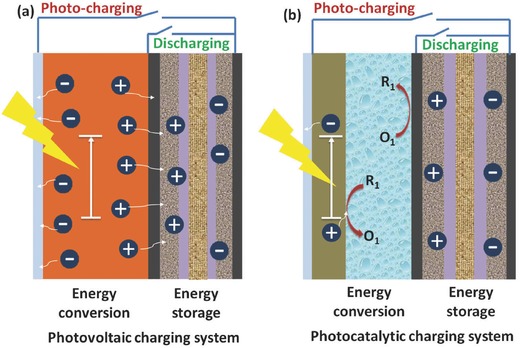
Schematic illustration of two groups of solar energy based IECSSs: a) Photovoltaic charging system and b) Photocatalytic charging system.

According to the different ways of energy conversion during the charging process, the solar energy based IECSSs can be divided into two groups as shown in **Figure** **1**. In the first group (Figure 1a), the energy conversion and storage units are normally separated and have independent electrochemical behaviour during the photo‐charging and discharging processes. There are no photo‐induced redox reactions during the photo‐charging process, which can be defined as photovoltaic charging system. For example, IECSSs based on most of the semiconductor solar cells such as silicon solar cells, organic solar cells and perovskite solar cells (PSCs), should fall into this group. The charging voltage on the energy storage part can be provided or partially provided by photovoltaic solar cells. In contrast, photo‐induced redox reactions will be involved during the energy storage (photo‐charging) process in a photocatalytic charging system. As illustrated in Figure 1b, during the photo‐charging process, the photoactive material on the photoelectrode will be excited to generate the electron hole pairs. The resultant electrons will then transfer to the energy storage electrode and the holes will be involved into the redox reactions and finally neutralized by the electrons from the counter electrode. During the discharging process, the charges will then come back to the counter electrode from the energy storage electrode, and the cations or anions in the electrolyte between these two electrodes will provide the counterbalance. In this section, recent progress and working mechanism of some typical representatives of the above two solar based IECSSs will be discussed. It should be noted that although the dye sensitized solar cells (DSSCs) have always been recognised as one kind of photovoltaic cell, the energy conversion in the DSSC is basically a photoelectrochemical process that involves photo‐induced redox reactions, which is different from the other semiconductor based solar cells. Therefore, here we place all the DSSC based IECSSs into the group of photocatalytic charging system.

### 2.1 Photovoltaic Charging System

**Figure 2:**
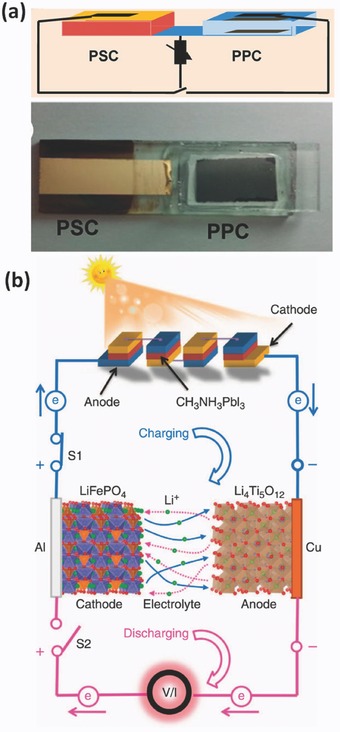
a) Schematic diagram of an IECSS containing a perovskite‐based solar cell (PSC) and a ploypyrrole based supercapacitor (PPC) (top), and photograph of the integrated system (bottom). Reproduced with permission.[[qv: 24a]] Copyright 2015, American Chemical Society. b) Schematic diagram of photo‐charging process of an IECSS consists of a LIB and four PSCs connected in series. Reproduced with permission.^23^ Copyright 2015, Nature Publishing Group.

In recent years, many types of integrated system with different photovoltaic cell units (i.e. silicon based solar cell,^21^ organic solar cells,^22^ PSCs^23^) and energy storage units (i.e. supercapacitors,^24^ LIBs,[[qv: 21c,23]] nickel metal hydride batteries[[qv: 21a]]) have been developed to realize the in situ storage of solar energy. The simplest way to integrate the energy conversion and storage units together is to connect them by wires.[[qv: 21a,c,23]] For example, Gibson and Kelly reported a combination of iron phosphate type Li‐ion battery and a thin amorphous Si solar cell. The integrated system achieved an overall solar energy conversion and storage efficiency of 14.5%.[[qv: 21c]] Later on, the same group used DC‐DC converter to elevate the low‐voltage PV voltage to over 300 V and charged the high‐voltage NiMH battery pack, resulting in an integrated system with a high solar to battery energy storage efficiency.[[qv: 21a]] Recently, the rapid development of high‐performance PSCs provides more opportunity for the development of not only highly efficient (up to ≈20%) low cost solar cells but also for the integration of solar cells into IECSSs for practical applications.[[qv: 10,24a,25]] For instance, an IECSS composed of a CH_3_NH_3_PbI_3_ based perovskite solar cell and a polypyrrole based supercapacitor was developed and shown in **Figure** **2**a.[[qv: 24a]] Solar energy can be converted and stored into the supercapacitor when they were in a parallel connection, while the stored energy can be discharged to help the solar cell achieve a high output power when they worked in series. The overall solar to electricity efficiency for the whole system was 10%. Soon after, the use of series‐connected CH_3_NH_3_PbI_3_ based PSC packs was reported for directly photo‐charging LIBs composed of a LiFePO_4_ cathode and a Li_4_Ti_5_O_12_ anode.^23^ As shown in Figure 2b, the photo‐generated holes and electrons within the PSCs will move to the LIB to realize the oxidation of the cathode and the reduction of the anode, respectively, leading to photo‐charging process. Then the stored energy can be released during the discharging process by switching S1 off and S2 on. The integrated PSC‐LIB system presented a high overall solar energy conversion and storage efficiency of 7.80% and excellent cycling performance.

**Figure 3:**
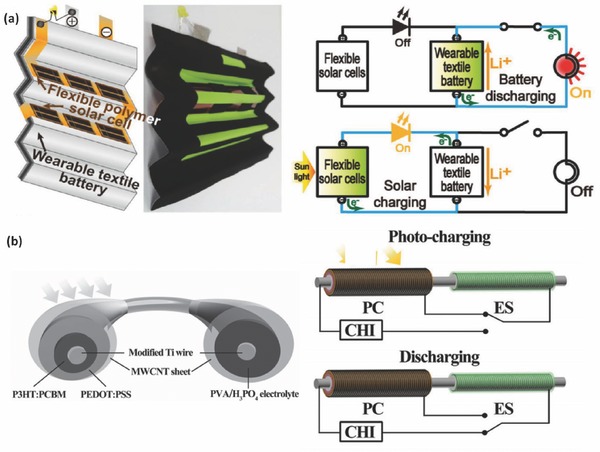
a) Schematic representation and photograph of the IECSS based on the textile battery and polymer solar cells (left) and equivalent circuits of the system in the photo‐charging and discharging processes (right). Reproduced with permission.[[qv: 22b]] Copyright 2013, American Chemical Society. b) Schematic illustration of the wire‐shaped IECSS composed of polymer solar cells for photoelectric conversion (PC) and supercapacitors for energy storage (ES) (left) and the equivalent circuits of the system in the process of photo‐charging and discharging. Reproduced with permission.[[qv: 28a]]

The wire‐connected IECSSs have many advantages in individual unit selection and assembly, however, the relatively long distance between the energy conversion and storage parts may lower the overall energy storage efficiency. One route to avoid this issue is to integrate the energy conversion part and the energy storage part into one device, which could also lead to space efficiency and then increase the volume energy density of the system. Many efforts have been devoted to develop such a system beginning with back‐to‐back design of the solar cells and batteries/capacitors (Figure 1a).^26^ These devices actually have similar working mechanism and individual energy conversion steps to the wire‐connected solar cell/battery system. Compared with other photovoltaic systems, organic solar cells are easy to be fabricated onto flexible substrates by various wet‐coating processes. Consequently, some flexible or wearable IECSSs based on organic solar cells have been developed in the past few years.[[qv: 22b,26,27]] For example, Lee et al. developed a wearable textile photo‐rechargeable battery with good mechanical stability by integrating an organic solar cell and a LIB (**Figure** **3**a).[[qv: 22b]] The battery was fabricated by covering the yarn with nickel layer and battery electrode material (Li_4_Ti_5_O_12_ or LiFePO_4_ for anode and cathode respectively), and then combined to a polymer solar cell with an energy conversion efficiency of 5.49%.[[qv: 22b]] In another case, CNT based capacitors was integrated with an organic solar cell (poly(3‐hexylthiophene (P3HT) with PCBM), forming a solid‐state photo‐capacitor.^26^ With the CNT coated aluminium layer as internal counter electrode, this system has a very thin (<0.6 mm) and light configuration, leading to a 43% reduction of device internal resistance as compared to the wire‐connected system.^26^

In addition to the back‐to‐back connected mode, many other photovoltaic charging system with special configurations such as wire or fibre shape^28^ and planar comb‐like structure,^29^ have been developed. For instance, a flexible “energy fibre” was designed by integrating the functions of photovoltaic energy conversion and storage.[[qv: 28a]] The authors designed the device with TiO_2_ nanotube modified Ti wire as core electrode and aligned MWCNT sheet as shell electrode. As shown in Figure 3b, the energy conversion unit was composed of P3HT:PCBM and PEDOT:PSS layers, while the energy storage part was made up of CNT and a poly(vinyl alcohol)/H_3_PO_4_ electrolyte. A overall solar energy conversion and storage efficiency up to 0.82% was achieved.[[qv: 28a]] Clearly, the integrated devices with both energy conversion and storage modules still have the challenging issue of how to better align the functions of two components to acheive higher conversion & storage efficiency.

### 2.2 Photocatalytic Charging System

**Figure 4:**
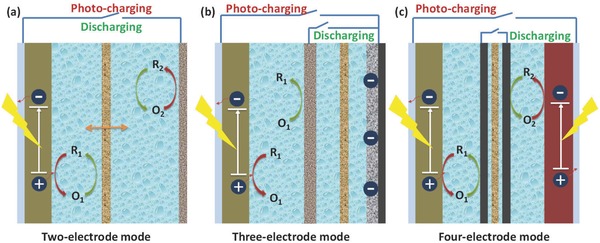
Schematic illustration of the photocatalytic charging systems with different electrode mode: a) Two‐electrode mode, b) Three‐electrode mode, and c) Four‐electrode mode.

Photocatalytic reactions have been intensively investigated and utilized in many solar energy conversion processes including DSSCs, solar‐hydrogen production, carbon dioxide reduction, etc.^30^ Particularly, over the past decade, great effort has been put into the integration of a DSSC and a capacitor/battery due to their similar structure and electrochemical nature.^3^ As a consequence, most of the reported photocatalytic charging systems are derived from a DSSC based configuration. Generally, the photo‐generated charges in photocatalytic charging system can be stored in many different ways, including double layer charges on electrode‐electrolyte interface, redox reactions of electrode materials, redox couples in the electrolyte, or a mixture of them. As illustrated in **Figure** **4**, several photocatalytic charging systems, including two‐electrode, three‐electrode and four‐electrode modes, have been developed with different electrode configuration and working mechanism, which will be introduced in this section.

#### 2.2.1 Two‐Electrode Mode

The first one shown in Figure 4a represents a two‐electrode mode, which has a similar structure to DSSC, and thus a same solar energy conversion process. The history of two‐electrode photocatalytic charging system can be traced back to 1980s.^31^ In the early stage, researchers tried to realize the energy storage function of photoelectrochemical cells by using multiple redox couples. For example, in 1981 Sharon et al. developed a two‐electrode system based on BaTiO_3_|Ce^4+/3+^||Fe^3+/2+^|Pt with two different types of redox couples separated by a film. During photo‐charging, one species (Ce^3+/4+^) would be oxidised on the surface of the photoelectrode and the other species (Fe^3+/2+^) would be reduced on the counter electrode. In this way, solar energy can be converted and stored in the battery, which can be further discharged in dark to produce electricity.[[qv: 31a]] Later on, Yonezawa et al. developed a photochemical storage battery with a configuration of n‐GaP|aqueous K_3_[Fe(CN_6_]−K_4_[Fe(CN)_6_||aqueous NiSO_4_|Pt, which can be charged under the photo irradiation.[[qv: 31c]] Stuart et al. also reported a solar rechargeable system with a structure of n‐Cd(Te, Se) photoelectrode|aquoues Cs_2_S_x_|SnS.[[qv: 31d]] However, the stability of the photoelectrode under light irradiation in these systems restricted their development for practical application.

**Figure 5:**
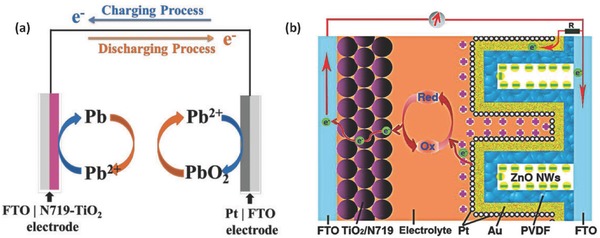
Representatives of two‐electrode photocatalytic charging systems. a) Schematic illustration of charging and discharging processes of solar Rechargeable Batteries based on lead‐organohalide electrolyte CH_3_NH_3_I·PbCl_2_. Reproduced with permission.^33^ b) An IECSS based on TiO_2_ DSSC with energy storage function by modifying the counter electrode with PVDF/ZnO nanowire array. Reproduced with permission.^36^

Much effort was made based on the dye‐sensitized TiO_2_ photoanode, which has been widely investigated for DSSC application. Selecting suitable redox couples in the electrolyte is an important strategy to realize the energy storage function for two electrode systems. For example, Hauch et al. proposed a two‐electrode photo‐charging battery consisting of dye‐sensitized TiO_2_ photoactive layer and WO_3_ coated conductive glass substrate as photoelectrode, a platinized conductive glass substrate as counter electrode, and propylene carbonate with Li^+^ and a redox couple I^−^ and I^3−^ as electrolyte. Under sunlight, the photo‐excited electrons will inject from the dye into the TiO_2_ and the holes will oxidise I^−^ to I_3_^−^. The electrons will diffuse from TiO_2_ to WO_3_, at which Li^+^ will then be intercalated into WO_3_ to keep the charge balanced. As a result, the solar energy is stored in the WO_3_|LiWO_3_||LiI|LiI_3_ redox system.^32^ In another case, Wang et al. demonstrated a new solar rechargeable battery by using a lead‐organohalide electrolyte CH_3_NH_3_I·PbCl_2_ (LOC) to replace the I^−^/I_3_^−^ electrolyte in conventional DSSC.^33^ As shown in **Figure** **5**a, under illumination, the photo‐generated electrons at the interface of the TiO_2_ electrode would reduce Pb^2+^ into Pb, and Pb^2+^ would be oxidized into PbO_2_ on the counter electrode. Consequently, charge is stored based on the Pb/Pb^2+^ and Pb^2+^/PbO_2_ redox couples. During the discharge, electrons will be released from the photoelectrode and pass through the external circuit to the counter electrode. Vanadium based redox battery with two‐electrode configuration have also been developed for solar energy storage. For instance, photocatalytic charging systems based on vanadium redox species were developed with FTO/TiO_2_ and FTO/TiO_2_/WO_3_ as the photoelectrodes and Pt mesh as the counter electrodes.^34^ Very recently, CdS thin film photoelectrode was introduced into a vanadium redox flow cell, yielding a photovoltage to charge the vanadium battery up to 75% with no external bias.^35^ However, the photoanodes in this system still need to be optimized due to the long‐term stability issue, and the selection of redox couples and electrolyte additivies are also of importance to further improve the coulombic efficiency of the energy storage modules in the IECSSs.

To improve the energy storage performance, efforts have also been made to explore new electrode system, which can store charges by electric charge double layer or redox reactions. For instance, a two‐electrode based photo‐rechargeable capacitor composing of dye‐sensitized TiO_2_ photoelectrode/hole‐trapping layer(LiI)/activated carbon was designed with an organic electrolyte solution. The photo‐generated charges at the electrode‐electrolyte interface can be stored as double layer charges directly on the active carbon surfaces.^37^ The photo‐capacitor shows an output voltage of 0.45 V after photo‐charging and yields a specific capacity of 75 mC cm^−2^ for discharge. In another case, rather than using traditional TiO_2_ as photoactive material, a two‐electrode mode system with a composite films composing of a conducting polymer and a dye as photoelectrode was developed.[[qv: 24b]] Structural modification of counter electrode has been also demonstrated helpful to achieve better performance. For example, Zhang et al. modified the counter electrodes of DSSCs with the high dielectric constant polyvinylidene fluoride (PVDF) and the high surface area ZnO nanowire array for the energy storage purpose (Figure 5b).^36^ Pt/Au particles were deposited onto the PVDF surface as a catalytic layer. With this design, the system can achieve concurrent power output and energy storage functions. The photo‐generated charges can move to Pt layer for photocatalytic process or to the surface of ZnO nanowires for energy storage. The Li^+^ will be adsorbed onto the Pt layer surface or diffuse into the PVDF layer for charge balance. The photo‐to‐electric conversion efficiency of the system could be up to 3.70%.^36^ In addition, photocatalysts can be also used as an electrode to assist the charge and discharge of different types of rechargeable batteries.^38^ For example, Liu et al. designed a photoassisted rechargeable Li‐O_2_ battery using g‐C_3_N_4_ photocatalyst as a photoelectrode to lower the charging voltage. This design can effectively address the overpotential problem of traditional Li‐O_2_ batteries and also promote the energy efficiency. Nevertheless, the selection of efficient and robust photocatalysts that have not only good light harvesting property but also excellent catalytic performance remain quite challenging for the devices.

#### 2.2.2 Three‐Electrode Mode

Although the two‐electrode mode system has advantages in device assembly and cost, a limit of this type is a high internal resistance hindering the discharging process as in most cases electrons have to go through the barrier at the semiconducting layer when going back to the photoelectrode. Thus, many new photocatalytic charging systems with various three‐electrode configurations (normally consisted of a photoelectrode, a counter electrode and an energy storage electrode) have been proposed, which can be further classified into two subgroups based on different functions of the counter electrode as shown in Figure 1b and 4b.

**Figure 6:**
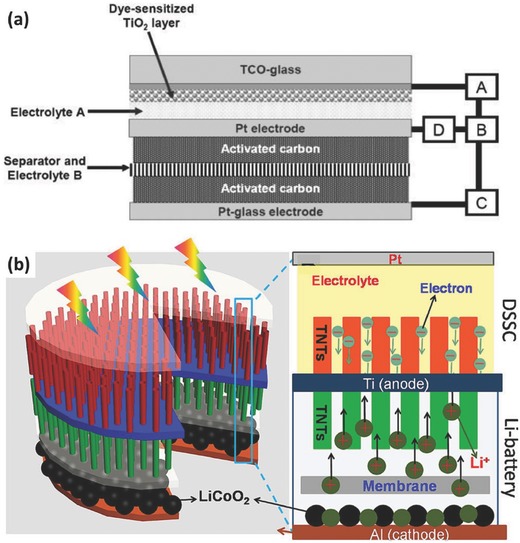
a) Structure illustration of one three‐electrode photocatalytic charging system. The photo‐capacitor consists of a dye‐sensitized TiO_2_ photoelectrode, a Pt‐plate counter electrode and a Pt‐coated glass charge storage electrode covered with activated carbon, spacer, separator, and electrolytes. Reproduced with permission.^39^ Copyright 2005, The Royal Society of Chemistry. b) Principle illustration of a three‐electrode photocatalytic charging system based on double‐sided TiO_2_ nanotube arrays. The top side of the Ti foil is used as photoelectrode in the DSSC part and the bottom part acts as anode part in a typical lithium ion battery for energy storage. Reproduced with permission.^44^ Copyright 2012, American Chemical Society.

In one case, the counter electrode is actually a double‐side electrode serving as a back‐to‐back connection between the separated photocatalytic energy conversion and storage system (Figure 1b). Under illumination, the photo‐excited electrons will travel through the external circuit to the charge storage electrode and reduce the active materials or store as electric double layer charge. Meanwhile, redox species will be oxidized on the photoelectrode and reduced on the counter electrode within the energy conversion part. The depleted electrons on the energy conversion side of the counter electrode will be balanced by the oxidation of the active material loaded on the other side. For example, Murakami et al. developed a photo‐capacitor with three‐electrode mode as shown in **Figure** **6**a.^39^ The bifunctional counter electrode serves for the electrolyte regeneration on one side and energy storage on the other. Later on, the research was expanded to solid^40^ or liquid state^41^ DSSCs and supercapacitors based on active carbon,^42^ carbon nanotubes,^26^ conductive polymer,^41^ TiO_2_ nanotubes,^43^ nickel oxide,^42^ and RuO_2_.[[qv: 40a]] Solar rechargeable lithium ion batteries with a similar three‐electrode configuration was reported by Guo et al.^44^ As shown in Figure 6b, this system is designed on the base of a Ti film with TiO_2_ nanotubes growing on double sides. The DSSCs part was fabricated with the dye‐sensitized TiO_2_ NT arrays as the photoanode and the battery part is composed of the TiO_2_ NT‐based anode and LiCoO_2_ cathode. During the photo‐charging process, the photo‐generated electrons will move into the conduction band of TiO_2_ from dyes and transport through the shared Ti film to the LIB anode part, which will further participate in the lithiation reaction of TiO_2_ anode. Meanwhile, delithiation of the LiCoO_2_ cathode materials will release free electrons that transfer to the counter electrode to balance the charges. As a result, this system delivered a discharge capacity of 38.89 µAh at a current density of 100 µA and showed an overall energy conversion and storage efficiency of 0.82%.^44^

Generally, the back‐to‐back type three‐electrode mode benefits from a smaller voltage drop, and higher overall energy conversion and storage efficiency.[[qv: 40a,44]] However, the separated energy conversion and storage system always has two individual energy conversion steps, which involves more potential drops, and thus no benefit for performance improvement.^45^ In another three‐electrode mode (Figure 4b), the counter electrode is designed as a shared electrode that can achieve the energy conversion and storage functions simultaneously.^46^ During the photo‐charging, photo‐generated charges move through the external circuit and stored at the energy storage electrode. The redox species in the electrolyte are simultaneously oxidised on the photo‐electrode by the holes and the solar energy is then stored. The photo‐charging and discharging process contains fewer energy conversion steps as compared to the above back‐to‐back separated system, potentially leading to higher solar energy storage efficiency and lower device cost. For example, Nagai and Segawa constructed a three‐electrode solar‐rechargeable battery with a Pt mesh as counter electrode by hybridizing a typical DSSC cell and a conducting polymer storage electrode.[[qv: 46a]] Solar energy was stored when the photo‐excited electrons reduce the polymer at the charge storage electrode and the redox species is oxidised (I^−^/I_3_^−^) into its oxidation state. This system afforded an energy density lower than 0.3 mW h cm^−2^ and a short self‐discharge time of no more than 10 min. Since then, a number of nanostructured materials such as CNTs,^47^ mesh carbon,^48^ conductive polymer,[[qv: 7c,49]] TiN nanotube arrays on the Ti mesh,^50^ have been explored in a good hope of replacing the expensive Pt counter electrode for energy storage, yet the performance/stability of these materials require further improvement.

**Figure 7:**
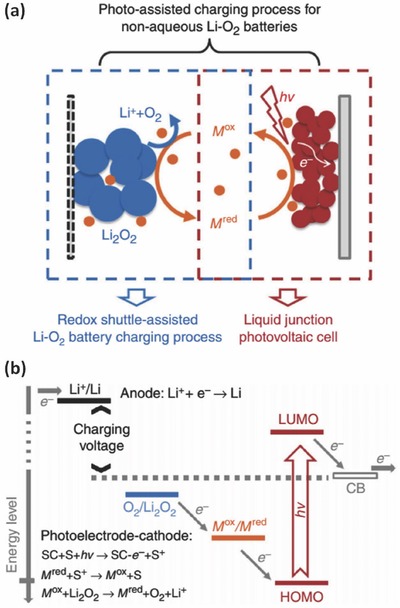
A three‐electrode photocatalytic charging system based on a lithium‐oxygen battery. a) The proposed mechanism and b) energy diagram of the solar battery system during the photo‐assisted charging process. (‘SC' stands for semiconductor and ‘S' stands for sensitizer.) Reproduced with permission.[[qv: 52a]] Copyright 2014, Nature Publishing Group.

Recently, development of new photocatalytic charging system on the base of other lithium based energy storage systems such as lithium ion battery,^51^ lithium‐oxygen batteries,^52^ lithium‐iodine batteries,[[qv: 46b]] and lithium‐sulfur batteries^53^ have been attracting increasing attention. For example, Yu et al. reported photo‐assisted charging of a lithium‐oxygen battery by coupling the photoelectrode and the oxygen electrode with a triiodide/iodide redox shuttle.[[qv: 52a]] The system was composed of a Li anode, an oxygen counter electrode and a dye‐sensitized TiO_2_ photoelectrode with non‐aqueous electrolyte. As shown in **Figure** **7**a, on photo‐charging, the redox species will be oxidized from its reduced form (M^red^) to M^ox^ on the photoelectrode and then diffuse to the Li_2_O_2_ loaded on the counter electrode. The Li_2_O_2_ will be oxidized into O_2_ and Li+ by M^ox^, which then will be reduced back to M^red^. With the help of the photocatalytic process, the system can be charged with a ‘negative' overpotential.[[qv: 52a]] Soon after that, a photo‐assisted charging process based on g‐C_3_N_4_ photocatalyst was successfully developed to address the overpotential issue of conventional non‐aqueous Li‐O_2_ batteries.[[qv: 52b]] Later on, Yu et al. extended this strategy to the metal‐iodine battery system.[[qv: 46b]] During the photo‐charging process, I^−^ ions can be photoelectrochemically oxidized into I_3_^−^, leading to a lower charging voltage (2.90 V) than that of conventional Li‐I batteries (3.30 V). As a result, an energy saving of close to 20% was achieved by the charging voltage reduction.[[qv: 46b]] Li et al. designed a photocatalytic charging system based on Li‐S battery in which the photo‐charging process can be achieved by photocatalytic oxidation of the discharging product (S^2−^) back to the polysulfide state.^53^ With a disconnected design between the photocatalytic part and the Li electrode, Li^+^ could not be reduced back to metallic lithium during the photo‐charging process but hydrogen can be photo‐generated with the aqueous electrolyte. As a result, this system delivered a discharge capacity of 280 mAh g^−1^ after two hours' light irradiation. A H_2_ generation rate of 1.02 mmol g^−1^ h^−1^ was achieved during the photo‐charging process as well.^53^ Note that the production of valuable hydrogen in the integrated systems is encouraging while its storage in an efficient and low‐cost manner remains a long‐standing challenge to the research community.

Three‐electrode photocatalytic charging redox batteries or RFBs have also been demonstrated for solar energy storage based on the redox couples in the anolyte and catholyte. For instance, a redox battery system was developed by Mahmoudzadeh et al. using S_4_^2−^/S_2_^2−^ and I_3_^−^/I^−^ as active species recently. This system exhibited an open‐circuit voltage of ≈0.7 V, which was close to the electrochemical potential difference of the redox couples.^45^ Yan et al. designed a solar rechargeable redox flow battery with organic compounds in aqueous electrolyte and LiI in organic electrolyte as anolyte and catholyte, respectively.^54^ Despite these successful examples, a main challenge for this type of system design is to select two pairs of redox couples that hold not only good solubility and electrochemical reversibility, but also suitable redox potentials for voltage output, which subseqently affect the conversion and storage performance of the intergrated systems.

#### 2.2.3 Four‐Electrode Mode

Four‐electrode based photocatalytic charging system is usually a combination of photocatalytic process with redox batteries or RFBs, in which a pair of redox couples will be employed as active species for energy storage.[[qv: 20b]] Particularly, RFBs have attracted great attention recently due to its advantages including long cycling life, high reliability, as well as relatively low maintenance costs, which are beneficial to large‐scale energy storage application.

**Figure 8:**
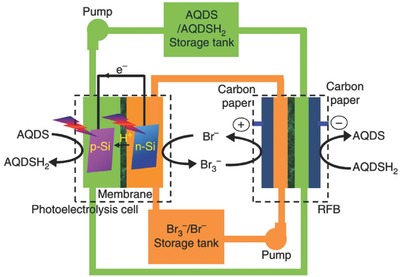
Schematic configuration of one four‐electrode photocatalytic charging system based on solar redox flow batteries, in which AQDS/AQDSH_2_ and Br_3_^−^/Br^−^ are used as redox couples. Reproduced with permission.^56^ Copyright 2016, Nature Publishing Group.

Several solar chargeable RFBs with four‐electrode design have been proposed in the past few years.^35^, ^55^ Liu et al. developed a photocatalytic charging RFB system with two electrolyte circuit loops, through which the redox species can enter the RFB unit for discharging separately, and then pump into the photocatalytic part for photoregeneration after discharge.[[qv: 55a]] Lithium‐ionic conducting films are required in both parts to separate the anodic and cathodic redox couples. The authors selected I_3_^−^/I^−^ and [Fe(C_10_H_15_)_2_]^+^/Fe(C_10_H_15_)_2_ (DMFc^+^/DMFc) as the redox couples due to their electrochemical reversibility and appropriate redox potentials. Further efforts are still required to improve the total energy conversion efficiency by structural design and materials optimization of the system.[[qv: 55a]] Very recently, Liao et al. reported a new solar rechargeable RFB system composing of a photoelectrochemical unit with two silicon photo‐electrodes and a quinone/bromine based RFB, which can convert solar energy into chemical energy and then into electricity, respectively.^56^ As shown in **Figure** **8**, AQDS/AQDSH_2_ (9,10‐anthraquinone‐2,7‐disulphonic sodium/1,8‐dihydroxy‐9,10‐anthraquinone‐2,7‐disulphonic sodium) and Br_3_^−^/Br^−^ were selected as active redox couples and separated by Nafion film in each unit. During the photo‐charging, AQDS was reduced and Br^−^ is oxidized on the photocathode and photoanode, respectively. The resultant AQDSH_2_ and Br_3_^−^ were then stored in individual reservoirs or pumped to the RFB for power output. With this design, an overall solar energy conversion and storage efficiency of up to 3.2% was achieved.^56^

## 3 Mechanical Energy Based IECSSs

Mechanical energy is another important energy source that can be converted into electrical energy to power electronics. Based on piezoelectric or triboelectric effects, various mechanical energies such as wind, waves, fluids etc. can be effectively converted into electricity that can be employed in powering nano‐devices.^6^, ^57^ In the past decade, many efforts have focused on the development of mechanical energy based IECSSs, and the progress on IECSSs related to piezoelectric effect will be reviewed as follows.

Piezoelectric effect is a unique property from piezoelectric materials that can deform under mechanical forces from the external environment to induce an electrical potential.[[qv: 12c]] Lead Zirconate titanate is a typical conventional piezoelectric material with high piezoelectric coupling and energy conversion rate, but it is too brittle to be shaped or minimized into desirable size.^58^ Moreover, the low electric conductivity of this material causes unidirectional Schottky barrier and poor electron transfer. Therefore, the conventional piezoelectric materials are difficult to satisfy the needs of the nano electronics and devices. In 2006, Wang et al. introduced the nano‐piezoelectric system based on aligned ZnO nanowire arrays.^59^ By coupling the piezoelectric and semiconducting properties of ZnO, the nanowire arrays can generate charge separation while bending and create an electrical current between the nanowire and the contacting metal tip. This approach successfully converted 17 to 30% of the mechanical energy into electricity, demonstrating great potential of developing nano‐piezoelectric systems that can harvest energy from the environment to power nano electronics. In general, nano‐piezoelectric system has some key advantages over traditional bulk piezoelectric materials. Firstly, their main physical and chemical properties including size, morphology, purity, composition and crystal structure can be precisely controlled and polished via lots of recently developed nanotechnology and nano synthesis methods. Secondly, the one‐dimensional nano architecture features a beneficial aspect ratio that can sensitively respond to a tiny force and generate a large potential gap. Based on these unique properties, some smart piezoelectric effect based IECSSs have been designed and intensively investigated.^4^, ^60^

**Figure 9:**
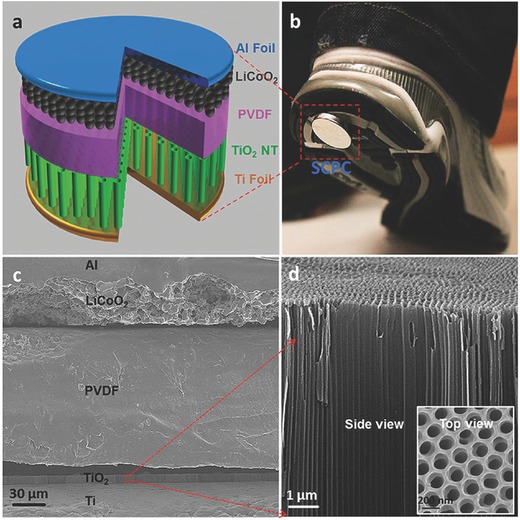
a) Illustration of the hybrid IECSS with a piezoelectric separator in a Li‐ion coin cell. b) A photo of the IECSS cell being attached to the bottom of a shoe. The cell will be charged by compressive energy from walking. c) Cross‐sectional SEM image of the cell. d) Enlarged view and top view (inset) of the red square in figure (c) showing Aligned TiO_2_ nanotubes. Reproduced with permission.[[qv: 60g]] Copyright 2012, American Chemical Society.

In 2012, Wang's group reported an IECSS that successfully realised the fundamental mechanism of directly hybridizing the energy conversion and energy storage processes in one unit.[[qv: 60g]] The system demonstrated the ability to harvests mechanical energy from the environment by piezoelectric effect and simultaneously recharge a Li ion coin cell. **Figure** **9** shows the principle design of the integrated system where a layer of polarized PVDF film replaces the traditional polyethylene (PE) separator. When an external force applies, the PVDF film will create a piezoelectric potential along its thickness, which will generate a local electric field and drive the directional diffusion of the Li ions. Thus the mechanical energy is converted into electricity and finally stored in the Li‐ion cell as chemical energy. However, as an early proof of concept device, it is difficult for practical applications mainly due to the non‐porous PVDF film that is unable to offer effective Li^+^ migration. To solve this problem, they modified the system recently by introducing a high degree of porosity into the PVDF film.^61^ Using ZnO particles as nucleating agents, β‐form piezoelectric PVDF was formed with good solubility and a lot of holes from the etching of ZnO particles. The new PVDF form provided a much lower charge transfer resistance and enhances the stability of the system; as a result, the new self‐charging power (SCPC) cell achieved 80% of charge/discharge capacity at 0.05 C, superior to only 10% in previous cells or 0% in cells using commercial PVDF film at the same rate. On the other side, making a composite film with PVDF and lead zirconate titanate (PZT) was also reported as a potential route to make practical SCPC recently.^62^ A charge capacity of 0.010 Ah in 240 s was achieved in SCPC with the PVDF‐PZT composite film while only 0.004 Ah can be generated in SCPC with pure PVDF film.

**Figure 10:**
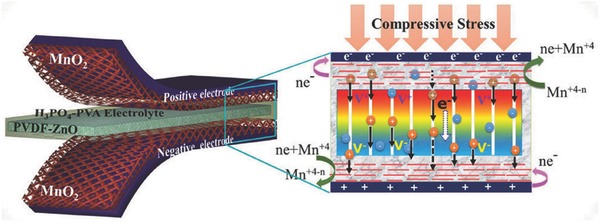
Schematic configuration of the SCSPC with symmetrical MnO_2_ electrodes and PVDF‐ZnO composite film separator. Enlarged part is the digital images of the piezoelectric effect and electrochemical reactions of the SCSPC under compressive stress. Reproduced with permission.^63^ Copyright 2015, American Chemical Society.

As another general energy storage device in nano‐electronics, supercapacitor has also been demonstrated to form a self‐charging supercapacitor power cell (SCSPC) by piezoelectric effect. Following the concept of using a piezoelectric effective separator (PVDF‐ZnO nanocomposite film), the SCSPC with MnO_2_ nanowires as both positive and negative electrodes was developed as shown in **Figure** **10**.^63^ The smartly designed cells showed the ability to be charged to 110 mV in 300 s by simply human palm impact, and more practically, illuminate green light‐emitting diode when serially connected. In another study in 2015, a flexible all‐solid‐state symmetrical supercapacitor was designed with polarized PVDF piezoelectric separator, H_2_SO_4_/poly(vinyl alcohol) gel electrolyte and functionalized carbon cloth electrodes.[[qv: 60f]] This device showed high capacitance under repeated bending and stretching and achieved a stable energy density of 49.67 mWh m^−2^ and a power density of 400 mW m^−2^ without rectification device, offering great potential as a self‐powering energy source for various nanosensors. In addition to the focus on using piezoelectric separators, a few other IECSSs with piezo‐electrochemical effect also attract recent attention. For example, a piezo‐electrochemical electrode using Li‐intercalated carbon fibres was reported to generate a potential of a few millivolts and be lithiated at a higher potential than delithiation when an external stretching force was applied.[[qv: 60a]] As a result, the mechanical energy was converted into electrochemical energy and leading to higher discharge energy of the electrode than charge energy. So far some simple mechanical energy based IECSSs have been demonstrated however a major challenge is the big voltage and/or current difference between generator output and the energy storage device required, which to a large extent limits the overall energy conversion and storage efficiency.

## 4 Thermal Energy Based IECSSs

Thermal energy is another abundantly available energy source, and most of it especially the low‐grade heat from such sources as industrial wastes, geothermal activity, and solar heating, is often wasted. Thermal‐electric energy conversion and storage has been demonstrated as an attractive technology to utilize this vast energy. Investigations in this field have focused on the exploration of solid‐state devices based on semiconductor materials for the conversion of thermal energy into electricity.[[qv: 5a]] The main problems with this way include the lack of energy storage capacity and the high cost semiconducting materials. Liquid‐based thermoelectrochemical cells (also known as thermogalvanic cells or thermocells) become an alternative due to the potential low cost design and scalablility.^64^ In thermoelectrochemical cells, the electrochemical potentials of redox couples (e.g., [Fe(CN)_6_]^4−/3−^ and Co^2+/3+^) are temperature dependent and can be used directly to generate a output voltage at different temperatures. The current research in this area are focusing on increasing the energy conversion efficiencies and output power and many strategies including selecting suitable redox couples[[qv: 60h,65]] and electrode materials,^66^ as well as the use of ionic liquids,[[qv: 65d,67]] have been proposed. However, only a few approaches were developed to endow thermoelectric system with energy storage capability.[[qv: 65c,68]]

**Figure 11:**
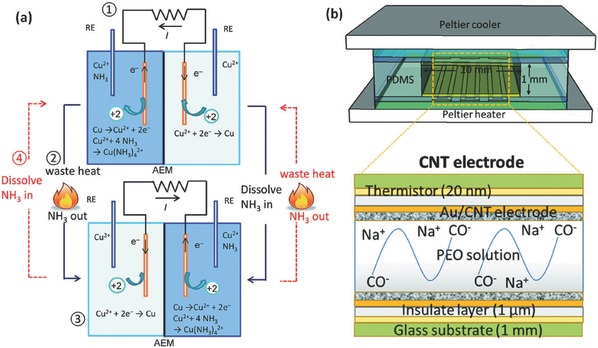
a) Schematic of the thermally‐regenerative ammonia‐based battery to convert waste heat into electricity. The system for harvesting waste heat include four steps: (1) power generation with one Cu^2+^ solution and another Cu^2+^ solution mixed with ammonia; (2) Regeneration of the electrolyte and NH_3_ by waste heat; (3) power production with regenerated electrolyte, during which the electrode is also regenerated; (4) regeneration of the electrolyte and NH_3_ by waste heat. Reproduced with permission.[[qv: 65c]] Copyright 2014, The Royal Society of Chemistry. b) Schematic of the ionic thermoelectric supercapacitor device with CNT electrodes and the reaction that occurs in the PEO solution. Reproduced with permission.[[qv: 68c]] Copyright 2016, The Royal Society of Chemistry.

Hartel et al. proposed a new heat‐to‐current converting system on the base of the temperature‐dependent property of the charged supercapacitors voltage at the charged state.[[qv: 68a]] The authors observed a thermal‐induced voltage rise of ≈0.6 mV K^−1^ within a temperature range of 0 °C to 65 °C for commercially available supercapacitors.[[qv: 68a]] Another sample is the recently developed thermally‐regenerative ammonia‐based battery (TRAB).[[qv: 60h,65c]] In a TRAB (**Figure** **11**a), both the cathode and anode were made of copper immersed in copper nitrate solutions. Ammonia (NH_3_) was added into the anolyte to charge the battery, resulting in a potential difference between the two electrodes. During discharge, the copper electrode was oxidized and dissolved into the NH_3_ solution, forming a copper ion ammine complex, while aqueous copper ions were reduced and deposited on the cathode. After discharging, electrolyte was regenerated by separating NH_3_ from the anolyte with waste heat. As a result, the waste heat energy can be stored through NH_3_ distillation and converted to electricity within the TRAB system.[[qv: 65c]] Based on this design, flow battery concept was further introduced into this system, overcoming some limitations of the previously reported system such as small electrode surface area.[[qv: 68b]] As a result, a much higher area power density (45 W m^−2^), volumetric energy density (1260 Wh m_anolyte_^−3^) were obtained than previous ammonia‐based systems.[[qv: 68b]]

Very recently, Zhao et al. reported a new thermoelectric system, which can convert the thermal energy into stored charge via a thermo‐diffusion of ions (Soret effect) in a polymeric electrolyte with a Seebeck coefficient as high as 10 mV K^−1^.[[qv: 68c]] As illustrated in Figure 11b, the thermoelectric liquid (PEO‐NaOH solution) electrolyte is sandwiched between two Au electrodes modified with self‐assembled multi‐wall carbon nanotubes, forming a supercapacitor for charge storage. At given temperature gradient, a Soret‐induced open thermovoltage can be obtained from non‐compensated thermos‐diffusion of cations and anions. Then thermal energy can be stored without the temperature gradient and discharged to an external circuit. The resulting thermoelectric system can convert and store much more energy than that of a traditional thermoelectric generator connected in series with a supercapacitor.[[qv: 68c]] This type of IECSSs shows their promosing aspects due to the widespread presence of thermal energy, however, the relatively low device efficiency and high cost of thermoelectric materials require much more R&D efforts towards their practical application.

## 5 IECSSs Based on Multi‐Type of Energies

In the above sections, we have introduced several types of IECSSs to collect the solar, mechanical, thermal energies, respectively. The development of more functional IECSSs that can scavenge and store multi‐mode energies from environment individually or simultaneously is more attractive but also highly challenging.^69^ Devices powered by this system can endure more complex environment by using whatever energy that might be available.

**Figure 12:**
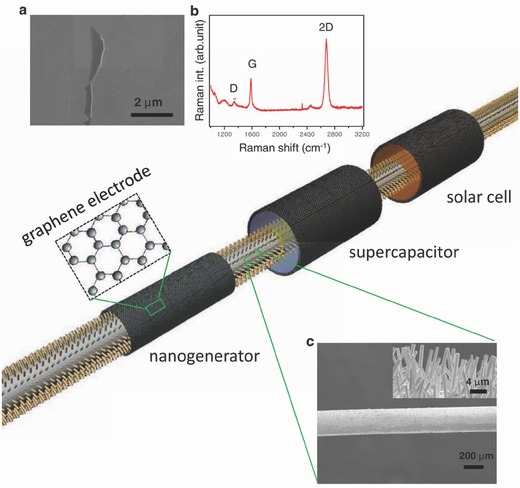
Schematic of a fiber‐shaped IECSS based on multi‐type of energies comprising of a nanogenerator, solar cell and supercapacitor. ZnO NWs are grown on a flexible thin plastic wire coated with thin Au film. High‐quality conductive and transparent graphene on Cu mesh were used as electrodes for each energy devices. For solar cell, and supercapacitor, the corresponding electrolytes were filled in between ZnO NWs and graphene electrodes. a) SEM image of a graphene film. b) Raman spectra of the graphene film. c) Low resolution SEM image of Au‐coated plastic wire covered with ZnO NW arrays. The inset is a SEM image of the plastic wire showing ZnO NW arrays grown along the radial direction. Reproduced with permission.^70^

There has been several reports on the development of the hybrid energy systems composed of more than one energy conversion units such as the piezoelectric, or triboelectric generators, thermoelectric generator and solar cells, as well as their integration with energy storage devices such as LIBs.^17^ For instance, Bae et al. reported a IECSS based on multi‐type of energies by integrating a nanogenerator, a DSSC as energy converter and a supercapacitor as storage device into one fiber‐shaped system (**Figure** **12**).^70^ In this system, the radially grown ZnO nanowires with large surface area can serve as active units for the nanogenerator that harvested mechanical energy[[qv: 57c]] and the active phase of the DSSC as well as the supercapacitor. Graphene was then employed as the cylindrical electrodes for all three parts due to its high conductivity and transparency. Such an energy system can harvest both solar and mechanical energy, and store it in a supercapacitor simultaneously, although the power and energy density still remains to improve.^70^

Another hybrid energy conversion system was developed with a thermoelectric generator at the bottom, a triboelectric nanogenerator at the middle, and a silicon based solar cell at the top.^71^ The designed system can be used to effectively capture solar, mechanical, and thermal energies simultaneously. Under the light illumination, the hybrid system showed output voltage and current of about 3.5 V and 30 mA, respectively. Under both the light and heat, the total voltage and current of the system can reach about 5.2 V and 34 mA, respectively. The collected energy by this system can be either stored in a LIB or used for water splitting.^71^

## 6 Conclusion and Perspective

In summary, this review provides an overview of the recent advances of IECSSs based on solar energy, mechanical energy, thermal energy, or multiple energies, which has been recognised as a promising way to simultaneously capture and store energy from the environment. Some of the key aspects and comparisons of these IECSSs are summarized in **Table** **1**.

**Table 1:** Comparison of key aspects of different IECSSs

IECSS	Solar energy‐based	Mechanical energy‐based	Thermal energy‐based	Multi energy‐based
Energy sources	Solar Energy	Wind, waves, fluids etc.	Heat from industrial wastes, geothermal activity, etc.	Combination of the solar, mechanical or thermal energies
Conversion device	Semiconductor solar cells, DSSC	Piezoelectric materials	Thermoelectric materials and devices	Multi‐conversion devices
Storage device	Supercapacitors; LIBs; hydride batteries	LIBs; Supercapacitors	Supercapacitors; TRAB	LIBs; supercapacitors
Typical integrated system configuration	Integrated Solar cell and LIBs; Integrated solar cell and supercapacitors	Piezoelectric or triboelectric materials integrated into LIBs or supercapacitors as multi‐functional separators	Liquid‐based thermoelectrochemical cells	Integrated LIBs with multi energy conversion devices
Typical overall efficiency	3.7 to 14.5%	N/A	0.1 to 5.7%	N/A

Currently, each type of the IECSSs has its unique features but also faces specific challenges. For the solar‐energy based IECSSs, one of the key advantages for integrated system is that it can save the energy loss of connecting the solar cells directly to the batteries by external wires. some organic solar cells can be even integrated into flexible or wearable IECSSs by various wet‐coating processes, which is very promising for the development of wearable self‐powered electronics. However, most of the research efforts focus on the design and conceptual demonstration of the integrated devices while limited effort is devoted to the overall efficiency optimization. For example, for some solar conversion systems such as photocatalytic processes, the solar conversion efficiency itself is still very low which requires much effort to achieve the threshold of commerical application. Another issue is that the solar components are generally difficult to match up perfectly with the batteries or supercapacitors considering the size difference and possible voltage mismatch, leading to poor electron transfer thus low efficiency. Consequently, it is important to develop more feasible devices with better integration of all the components in the systems. For example, wire‐shaped parts can match up better with other components and achieve higher efficiency. The four‐electrode design, such as the RFBs described above, is another good example because the energy conversion and storage components can be optimized independently. Mechanical‐energy based IECSSs are important for the development of medical devices, personal electronics, etc., because the devices can be self‐powered from using the mechanical energies from the environment such as the movement of human body. In addition, the integrated system can effectively promote the overall efficiency than directly connecting the two separated units. However, this type of IECSSs is still in the early stage and most of the works focus on the conceptual demonstration, but the performance of both the energy harvesting and conversion components are still quite low. For example, it was observed that the saturation voltage of the energy storage unit is much smaller than the voltage that the nanogenerators can provide.^72^ This will significantly decrease the energy storage efficiency. In addition, the charging rate for the storage unit decreased very quickly, which is also possibly due to the voltage mismatch issue. For the future applications of mechanical energy‐based IECSSs, more mechanism understanding of the energy harvesting and charge transfer in the integrated system is necessary to guide the enhancement of both functional modules on performance including the efficiency, stability etc. On the other side, it is also important to explore new materials and system configurations to improve the performance and decrease the cost for possible scale‐up production. Thermal‐based IECSSs propose a potentially practical way of converting low‐grade heat energy effectively into electricity. And the demonstrated example of TRAB system shows a significantly higher power density than salinity gradient energy technologies.[[qv: 65c]] The other reported thermal conversion systems coupled with supercapacitors also claimed the potential of scale‐up productions for practical heat‐to‐current applications because they use cheap and abundant materials.[[qv: 31c,68a]] Although this type of IECSSs show their promosing aspects, the development of thermal conversion materials still faces significant challenges including the high cost of materials, low thermal stability and quite low efficiency. In addition, the design and concept of these IECSSs is novel while the working principle of each component is still unclear. Therefore, more systematic understanding on the heat‐to‐current transfer process of these devices will be essential to improve the efficiency. Another urgent need is that new materials and device configurations should be further investigated to meet the cost and performance requirement of practical scaleable IECSSs. Multi‐energy based IECSSs can convert different types of energy and fit more environment circumstances. Basically, the concept is to physically integrate two energy conversion units with the storage unit. However, considering the large difference of each unit like the configuration and working environment, it can be much more difficult than a single source IECSSs. Despite a few recent conceptual breakthrough reports, the multi‐energy IECSSs still have a long way to go for the practical applications before their cost, efficiency and stability can be substantially improved.

Although IECSSs remain relatively less explored than individual devices and there still a long way from practical application, the IECSSs research over the past few decades have proved to be highly effective and promising with many conceptual demonstrations at a lab‐scale level. As a multi‐interdisciplinary research field, further achievements on IECSSs would be continuing to attract research efforts from both the energy conversion and storage fields. More specifically, from the viewpoints of materials selection and system configuration, future works on the following directions are highly expected.

(i) Most of the current investigations on IECSSs are still focused on the design and demonstration of prototypes. The overall energy conversion and storage efficiencies of these devices are always lower than those of the isolated counterparts. As the overall efficiency of the system is dependent on both the conversion and storage processes, high performance active materials for both parts are required to achieve desired overall system performance, which should be the mainstream at present stage. (ii) Rational design of system configuration and development of reliable packaging technologies are critical important to improve the overall energy efficiency and adapt to future practical applications. For example, exploration of high performance flexible and wearable IECSSs for powering future flexible electronics will be one important research direction in this area. (iii) Exploration of new conceptual energy conversion or storage devices and design of relevant integrated system would be more interesting and challenging. Considering the fast development of new energy conversion and storage technologies, as well as integrated system fabrication techniques, it is reasonable to believe that the IECSSs will become an appealing practical approach for energy conversion, storage, and distribution in the near future.

## Conflict of Interest

The authors declare no conflict of interest.
